# DEMENTIA-FRIENDLY IN THE CONTEXT OF HOSPITALIZATION: A CONCEPT ANALYSIS

**DOI:** 10.1093/geroni/igac059.2816

**Published:** 2022-12-20

**Authors:** Ellen Munsterman, Pamela Cacchione, Nancy Hodgson, G Adriana Perez

**Affiliations:** University of Pennsylvania, Philadelphia, Pennsylvania, United States; University of Pennsylvania, Philadelphia, Pennsylvania, United States; University of Pennsylvania, Philadelphia, Pennsylvania, United States; University of Pennsylvania, Philadelphia, Pennsylvania, United States

## Abstract

Persons living with dementia experience more and longer hospitalizations when compared to those without dementia. In response, initiatives to address the hospital experience for individuals with dementia and their families are becoming more common. The term “dementia-friendly” attached to terms such as “hospital”, “ward”, “initiative”, “program”, etc. is being used to describe this response. Literature has begun to summarize current knowledge of dementia-friendly initiatives and define characteristics of dementia friendly hospitals, however, a formal definition of the concept of “dementia-friendly” is lacking. A concept analysis was completed using the 8-step Walker and Avant method including: selecting the concept and aim, identifying use of the concept in the literature, determining defining attributes, antecedents, consequences, and empirical referents, and developing cases that further distinguish the concept. The goal of this analysis was to clarify the definition of dementia-friendly in the context of hospitalization. Attributes of dementia-friendly in the hospital include dementia-specific staff education, use of person-centered care practices, environmental modification, and nursing-specific factors such as attitude, time to spend with patients and adapting responses based on patient need. Antecedents include recognition of the impact of hospitalization on older people with dementia. Consequences include enhanced staff knowledge and patient outcomes including decreased length of stay and decreased use of antipsychotic medication. Based on the above analysis, a conceptual definition for dementia-friendly in the context of hospitalization is proposed. This definition highlights the identified antecedents and attributes with a focus on the essential role of nursing in delivering dementia-friendly care.

